# The abdominal wall hernia in cirrhotic patients: a historical challenge

**DOI:** 10.1186/s13017-018-0196-z

**Published:** 2018-07-28

**Authors:** Giuseppe Salamone, Leo Licari, Giovanni Guercio, Sofia Campanella, Nicolò Falco, Gregorio Scerrino, Sebastiano Bonventre, Girolamo Geraci, Gianfranco Cocorullo, Gaspare Gulotta

**Affiliations:** Department of Surgical, Oncological and Oral Science, University of Palermo, Policlinico P. Giaccone. Via Liborio Giuffré 5, 90127 Palermo, Italy

**Keywords:** Abdominal wall hernia, Cirrhosis, Surgery, Emergency, Risk factors

## Abstract

**Background:**

The incidence rate of abdominal wall hernia is 20–40% in cirrhotic patients. A surgical approach was originally performed only if complication signs and symptoms occurred. Several recent studies have demonstrated the usefulness of elective surgery. During recent decades, the indications for surgical timing have changed.

**Methods:**

Cirrhotic patients with abdominal hernia who underwent surgical operation for abdominal wall hernia repair at the Policlinico “Paolo Giaccone” at Palermo University Hospital between January 2010 and September 2016 were identified in a prospective database, and the data collected were retrospectively reviewed; patients’ medical and surgical records were collected from charts and surgical and intensive care unit (ICU) registries. Postoperative morbidity was determined through the Clavien-Dindo classification. Cirrhosis severity was estimated by the Child-Pugh-Turcotte (CPT) score and MELD (model of end-stage liver disease) score. Postoperative mortality was considered up to 30 days after surgery. A follow-up period of at least 1 year was used to evaluate hernia recurrence.

**Results:**

The univariate and multivariate analyses demonstrated the unique independent risk factors for the development of postsurgical morbidity (emergency surgery (OR 6.42; *p* 0.023), CPT class C (OR 3.72; *p* 0.041), American Society of Anesthesiologists (ASA) score ≥ 3 (OR 4.72; *p* 0.012) and MELD ≥ 20 (OR 5.64; *p* 0.009)) and postsurgical mortality (emergency surgery (OR 10.32; *p* 0.021), CPT class C (OR 5.52; *p* 0.014), ASA score ≥ 3 (OR 8.65; *p* 0.018), MELD ≥ 20 (OR 2.15; *p* 0.02)).

**Conclusions:**

Concerning abdominal wall hernia repair in cirrhotic patients, the worst outcome is associated with emergency surgery and with uncontrolled disease. The correct timing of the surgical operation is elective surgery after ascites drainage and albumin/electrolyte serum level and coagulation alteration correction.

## Background

The overall incidence of abdominal wall hernias is approximately 14%; it increases to 20% in cirrhotic patients and might be up to 40% in cases of major ascites [1, 2]. Factors such as weakness of the fascia and of the abdominal muscles due to malnutrition state and enlargement of pre-existing openings in the fascia promoted by increased abdominal pressure as a result of ascites formation are important contributors to the development of the hernias [3, 4]. The watch-and-wait policy was commonly accepted in the past because of the high perioperative morbidity and mortality that cirrhotic patients encountered. A surgical approach was then performed only if complication signs and symptoms occurred. However, the recommendations have changed during the last decade [5–7]. Previous retrospective studies [8] demonstrated that conservative treatment of abdominal wall hernias in cirrhotic patients is associated with considerable morbidity and mortality. Optimizing the patients with liver cirrhosis before elective hernia repair is critical for minimizing postoperative complications and reducing recurrence.

Moreover, it is commonly accepted that abdominal wall hernia repair should ideally be performed during liver transplantation or during liver function improvement.

In candidates for liver transplantation, the surgical operation should be performed during transplantation unless the patient presents with significant symptoms or hernia complications or if the perspective to be transplanted exceeds 3–6 months [9].

Several studies have demonstrated that elective surgery in cirrhotic patients could be safe, even when refractory ascites or advanced cirrhosis is diagnosed, if it is performed in a high-volume liver center [9–11]. Early elective hernia repair in these patients should be advocated considering the hepatic reserve and the patient’s condition. The study herein analyzed the characteristics of cirrhotic patients who underwent abdominal wall hernia repair and investigated the risk factors for postoperative morbidity and mortality.

## Methods

Cirrhotic patients with abdominal hernia who underwent surgical operation for abdominal wall hernia repair at the Policlinico “Paolo Giaccone” at Palermo University Hospital between January 2010 and September 2016 were identified in a prospective database, and the data collected were retrospectively reviewed; patients’ medical and surgical records were collected from charts and the surgical and ICU registries.

The diagnosis of abdominal wall hernia was obtained after physical examination and US/CT scan execution.

Cirrhosis was documented by anamnestic data and confirmed through clinical, laboratory, and radiological findings.

Postoperative morbidity was determined through the Clavien-Dindo classification; classes III to V events were considered major complications. Cirrhosis severity was estimated by the Child-Pugh-Turcotte (CPT) score and MELD score calculated at the time of the surgical procedure.

Postoperative mortality was considered up to 30 days after surgery [12–14]. A follow-up period of at least 1 year was used to evaluate hernia recurrence, as diagnosed with physical examination and US/CT scan.

Patients with refractory ascites underwent paracentesis, albumin and serum electrolytes were replaced, nutritional support was guaranteed, and coagulation disorders were corrected pre- and postoperatively when indicated.

Abdominal wall hernias were repaired with the direct suture repair surgical technique when the defect did not exceed 3 cm or in cases of a contaminated/dirty surgical field; otherwise, the mesh-repair technique was adopted with sublay retromuscular positioning of a polyester mesh fixed at the posterior fascia of the rectus abdominis muscle with non-reabsorbable sutures. Indirect inguinal hernias were repaired with the plug- and mesh-mediated technique using a polypropylene plug fixed in the internal inguinal ring at the conjoint tendon and Cooper ligament; the polypropylene mesh was then positioned under the aponeurosis of the external oblique muscle (Trabucco technique). Direct inguinal hernias were repaired by performing the Lichtenstein technique after sac isolation and inversion into the preperitoneal space, preserving the integrity of the peritoneal sac. Patients with a diagnosis of recurrent abdominal wall hernia were not enrolled in the database. All surgical operations were performed via laparotomy.

Patient characteristics are shown in Table 1.

**Table 1:** Characteristics of the population

Variables	Population (*n* = 117)	Elective surgery (*n* = 76)	Emergency surgery (*n* = 41)
Age	60 (53–81)	60 (53–81)	65 (60–81)
Male	100 (86%)	66 (88%)	34 (82%)
Mean BMI	25	27	24
Poorly controlled ascites (*n* of patients)	41	14	27
CPT: A (*n* of patients)	41 (35%)	41 (54%)	0
B (*n* of patients)	27 (23%)	23 (30%)	4 (10%)
C (*n* of patients)	49 (42%)	12 (16%)	37 (90%)
Mean pre-operative MELD score	13	12	16
n of patients with MELD score ≥ 20	32 (27%)	15 (20%)	17 (41%)
Mesh use	76 (65%)	55 (47%)	21 (18%)
Mean in-hospital stay (days)	10	7	16
Post-operative ICU (*n* of patients)	23 (20%)	8 (10%)	15 (36%)
Mean ICU stay (days)	7	2	10
Death	27 (23%)	5 (6.6%)	22 (53.6%)
Clavien-Dindo score I	27	27	0
II	22	22	0
III	31	22	9
IV	10	0	10
V	27	5	22
ASA score < 3	29 (25%)	29 (38%)	0 (0%)
ASA score ≥ 3	88 (75%)	47 (62%)	41 (100%)
Bilateral hernia	6	4	2
Mesh use	6	4	2
General anesthesia	1 (20%)	0	1
Spinal anesthesia	5 (80%)	4	1
Monolateral hernia	30	19	11
Mesh use	30	19	11
General anesthesia	1 (3%)	0	1
Local anesthesia	26 (87%)	16	10
Spinal anesthesia	3 (10%)	3	0
Umbilical hernia	60	37	23
Mesh use	19	16	3
General anesthesia	3 (5%)	0	3
Local anesthesia	57 (95%)	37	20
Incisional hernia	21	16	5
Mesh use	21	16	5
General anesthesia	21 (100%)	16	5

Twenty-six patients (22%) were operated on under general anesthesia; 83 patients (71%) underwent the surgical operation under local anesthesia, and eight patients (7%) had spinal anesthesia. Details regarding the distribution of the type of hernia, anesthesia regimen used for each surgical operation and mesh usage are shown in Table 1.

### Statistical analyses

Data were analyzed using Excel 2013 and IBM SPSS software, version 21. The median was obtained for continuous variables. Comparisons of continuous variables were made using Student’s *t* test or the Mann-Whitney test, where appropriate. Comparisons of categorical variables were made with the chi-squared (χ^2^) test or Fisher’s exact test. The statistical significance level was set to *p* value < 0.05.

Univariate analysis for morbidity and survival was performed; the clinical variables included were emergency, CPT, ASA score, ascites, prosthesis use, MELD score, age, sex, and general anesthesia; the type of hernias was evaluated in the univariate analysis to identify possible risk factors for postoperative morbidity. The variables with *p* values < 0.05 in the univariate analysis were included in the multivariate logistic regression, considering odds ratios with 95% confidence intervals and *p* values < 0.05.

## Results

Between January 2010 and September 2016, 117 cirrhotic patients were identified as undergoing abdominal wall hernia repair. Forty-one patients (35% of the cirrhotic patients with abdominal wall hernia) were treated in emergency situations. The median pre-operative MELD score was 13. The MELD score rate ≥ 20 in elective and emergency surgery was 20% and 41% respectively (Table 1). The minimal follow-up time was 1 year. Mesh positioning was performed in 76 cases, of which 21 were in the emergency group. Emergency criteria were perforation (*n* = 4), incarceration (*n* = 27), strangulation (*n* = 7), and skin ulceration (*n* = 3).

Six patients had bilateral inguinal hernia, 30 had monolateral inguinal hernia, 60 had umbilical hernia, and 21 had incisional hernia.

Death occurred in 27 patients within 30 days after surgery and in 22 after emergency surgical operations; the causes of death were MOF due to sepsis after infection of the ascites (*n* = 16) and major ascites for decompensated cirrhosis (*n* = 6) with ascites leakage. In the elective group, death occurred after postoperative heart attack (*n* = 1), major ascites for decompensated cirrhosis (*n* = 2), and acute kidney failure (*n* = 2).

Emergency patients also presented with a markedly higher number of perioperative class III–V complications according to the Clavien-Dindo classification.

The median in-hospital stay was 10 days. Longer median hospital (16 vs. 7 days) and intensive care unit (10 vs. 2 days) stays were observed in the emergency patient group. Two hernia recurrences (monolateral indirect inguinal hernia—P2L according to European Hernia Society (EHS) classification—treated with Trabucco mesh repair and umbilical hernia treated with direct suture repair) were identified in the emergency group during the follow-up period; the umbilical hernia was then electively treated with the mesh-mediated pre-peritoneal open technique; the recurrent inguinal hernia was then electively treated by identifying the sac and the defect. The defect was diagnosed in the canal’s posterior wall, describing a direct recurrent inguinal hernia, perhaps from displacement of the mesh from the pubic tubercle surface, where it was first anchored; a plug was then positioned in the defect after hernia inversion inside the abdominal cavity, reconstructing the anatomy of the inguinal canal.

The univariate analysis conducted to identify if the type of hernia could represent a risk factor for postoperative morbidity showed a considerable *p* value for bilateral inguinal hernia (*p* < 0.01) and incisional hernia (*p* < 0.01) (Table 2) not confirmed on the multivariable analysis. In the same way, we demonstrated that general anesthesia had a considerable *p* value in the univariate analysis for morbidity (*p* = 0.009) and mortality (*p* = 0.008) not confirmed on the multivariable analysis (Table 2).

**Table 2:** Univariate and multivariate logistic regression analyses for morbidity

	OR	95% CI	*p*
Univariate			
Emergency	11.62	3.23–40.76	0.026
CPT C	10.49	2.16–32.43	0.003
ASA score ≥ 3	9.43	2.01–25.57	0.001
Ascites	0.76	0.23–50.78	0.114
Mesh use	0.54	0.57–70.87	0.214
MELD score ≥ 20	2.06	2.41–22.76	0.017
Age > 60	1.53	0.12–56.19	0.421
Male	1.54	0.09–12.93	0.769
Bilateral inguinal hernia	7.62	5.32–30.11	0.009
Monolateral inguinal hernia	3.23	0.82–11.41	0.11
Umbilical hernia	0.76	0.98–45.71	0.23
Incisional hernia	1.82	2.92–7.34	0.009
General anesthesia	4.32	2.76–80.91	0.009
Multivariate			
Emergency	6.42	1.76–40.53	0.023
CPT C	3.72	1.23–37.28	0.041
ASA score ≥ 3	4.72	3.41–45.81	0.012
MELD score ≥ 20	5.64	1.71–23.67	0.009
Bilateral inguinal hernia	4.12	0.42–46.71	0.17
Incisional hernia	6.75	0.67–52.86	0.26
General anesthesia	2.87	0.12–34.22	0.12

The variables with *p* values < 0.05 in the univariate analysis were included in the multivariate logistic regression. The multivariate analysis conducted showed that emergency surgery, CPT class C, ASA score ≥ 3, and MELD ≥ 20 were unique independent risk factors for the development of postsurgical morbidity (Table 2) and mortality (Table 3).

**Table 3:** Univariate and multivariate logistic regression analyses for mortality

	OR	95% CI	*p*
Univariate			
Emergency	21.76	4.26–31.53	0.003
CPT C	3.56	13.21–76.32	0.001
ASA score ≥ 3	10.31	3.54–16.32	0.001
Ascites	2.45	0.22–67.21	0.51
Mesh use	1.21	0.86–14.32	0.41
MELD score ≥ 20	1.69	2.02–23.63	0.017
Age > 60	2.47	0.63–45.61	0.21
Male	0.12	0.03–10.45	0.53
General anesthesia	2.43	5.28–43.61	0.008
Multivariate			
Emergency	10.32	3.66–47.82	0.021
CPT C	5.52	1.67–32.45	0.014
ASA score ≥ 3	8.65	3.65–87.23	0.018
MELD score ≥ 20	2.15	2.71–32.68	0.002
General anesthesia	7.22	4.71–13.65	0.23

## Discussion

Traditionally, hernia repair in the presence of advanced cirrhosis and ascites has resulted in high rates of morbidity and mortality, prompting many surgeons to avoid elective repair and to operate only when complications develop. In 1960, Baron reported a mortality of 31% in a case series of 16 patients who underwent umbilical hernia repair and who had cirrhosis. O’Hara et al. reported a morbidity rate of 22% and a mortality of 16% in emergency surgery; these data suggest that surgical repair should be performed in uncomplicated hernias. The risk of treating complicated hernia conservatively heavily outweighs the risk of surgical repair. Non-operative management of complicated hernias with antibiotics and dressing changes might result in mortality rates in the range of 60–88%. Therefore, complicated umbilical hernias in cirrhotic patients should be repaired emergently [15].

The complications related to surgical operations of abdominal wall hernias are high in cirrhotic patients, as much of the impact of abdominal hernia presence is on QoL. In recent decades, the indications for surgical timing and management have changed. The watch-and-wait strategy has been abandoned in favor of elective surgery. However, the indications for surgical repair of abdominal wall hernias in cirrhotic patients remain a controversial challenge.

Expectant treatment of cirrhotic patients with abdominal wall hernia and ascites is associated with an increased rate of complications, such as incarceration, evisceration, ascites drainage, and peritonitis. These complications require emergency surgical treatment, which carries increased risk of morbidity and mortality. Conversely, elective hernia correction might be performed with fewer complications and is therefore advocated [9].

It has been demonstrated that the improved complication rates associated with modern surgical techniques and perioperative care justify the consideration of an early repair before complications occur. Kirkpatrick and Schubert reported improved outcomes and lower mortality in patients treated after 1975 than in patients treated prior to 1975. A review by Maniatis and Hunt of papers published between 1956 and 1990 found a mortality rate of only 2% in the non-emergency setting, whereas the mortality rate was 14% with repair due to complications carried out as an emergency [16].

The data proposed suggest that emergency, CPT-C, ASA score ≥ 3, and MELD score ≥ 20 are risk factors for postoperative morbidity and mortality. In contrast, elective surgery appears to be successful and to be associated with lower mortality rate. Scientific reports indicate that adequate preparation of cirrhotic patients, with control of ascites, albumin and electrolytes serum levels, nutritional support, and coagulation patterns, allows for the success of elective surgery [16, 17].

The mortality rates for elective and emergency patients reported by our series were, respectively, 6.6 and 53.6%. A reported mortality rate higher than that in the data published in the international literature can be explained not only by the high rate of MELD score ≥ 20 in the two groups (20 and 41%, respectively), but also by the high rate of CPT class C and ASA score ≥ 3 that are respectively 16 and 62% in elective surgery group, 90 and 100% in emergency surgery group, as reported in Table 1.

Ascites control is essential to reducing perioperative complications and recurrence.

In the past, there was a considerable lack of evidence regarding how severe liver dysfunction must be to preclude operative repair. There did not appear to be any reliable, commonly accepted methods to determine whether the cirrhosis was too severe to allow for elective repair or was mild enough that the risk of major complications was low enough to justify the repair [16].

It has now been demonstrated that the CPT score and MELD score are the best ways to identify the severity of liver illness; these scores adequately correlate with prognostic evaluations of postoperative morbidity and mortality in cirrhotic patients.

Recently, although the MELD score was optimized for liver transplantation patients, it appears to be the most objective means to evaluate the surgical risk in cirrhotic patients. It has been demonstrated that a MELD score between 8 and 14 predicts poor surgical outcomes. Moreover, the worst outcome for abdominal surgery is described when the MELD score is above 20 [18–26]. The median pre-operative MELD score in our series was 13.

According to the multivariate analysis, elective surgery is preferable concerning the timing for hernia repair. Emergency surgery is strongly associated with a higher incidence of postoperative morbidity and mortality.

Refractory ascites is frequently associated with urgency even if it does not represent a risk factor in the multivariate analysis. Refractory ascites is surely considered a direct cause of a complicated hernia because of the increased abdominal pressure. This complication is also correlated with skin ulceration, risk of SSI, and ascites leaks.

## Conclusions

The results of the data analysis show that performing the surgical operation of abdominal wall hernia repair in cirrhotic patients emergently is related to higher postoperative morbidity and mortality rates. This finding suggests that the correct timing of the surgical operation is elective surgery in controlled liver disease, monitoring the disease with the CPT score and the MELD score. An ASA score ≥ 3 is also a risk factor for postoperative morbidity and mortality. All these risk factors should be considered in the prognostic evaluation of cirrhotic patients who require surgical operation for abdominal wall hernia repair. Furthermore, pre-operative refractory ascites should be managed with paracentesis and albumin/electrolytes serum level and coagulation alteration with appropriate correction.
